# A Lecture on Caries and Necrosis of Bone

**Published:** 1868-01

**Authors:** H. R. Noel

**Affiliations:** Professor of Physiology.


### AMERICAN JOURNAL

OF

### DENTAL SCIENCE.

Vol. I. THIRD SERIES--JANUARY, 1868. No. 9.

### ORIGINAL COMMUNICATIONS.

### ARTICLE I.

### *A Lecture on Caries and Necrosis of Bone,*

Delivered Nov. 15, 1867, at the Baltimore College of Dental Surgery,

By H. R. Noel, M.D., Professor of Physiology.

Gentlemen :

We have before us to-day, the very delicate operation of critical analysis ; we wish to examine carefully and minutely Dr. L. S. Beale's Theories of Caries and Necrosis of Bone. We have given you his views of the *structure* and *developement* of teeth and of bone, that is, his physiology.

Dr. Beale's Physiology is so far good—very good ; but his Pathology is bad, very bad,—his Aetiology is worse than bad—it is false.

A single glance at the anatomy of bone and we will begin the analysis.

You remember the Ossicle or Haversian system ; the canal of Havers ; the concentric laminæ :—the concentric lacunæ ; the radiating canaliculi. You remember these as characteristices of *dry bone,* but recent bone has a bloodvessel in the canal of Havers—germinal matter in the lacunæ—and nutrient fluid or pabulum in the canaliculi. Hence a nutritious fluid circulates in every direction through bone.

Now here is "Dr. Beale's Theory of Caries" as given in his work called—Structure, Life and Growth, pp. 145.

"In *Caries* the "germinal matter" (in the lacunæ) of a part of a bone receives too large a supply of nutrient matter, it grows too fast and lives upon the surrounding tissue which has been already formed."

This, taken from the same work, is his Theory of Necrosis.

"In necrosis the death of the germinal matter of many lacunæ takes place. It is easy to conceive that such a result must ensue if the supply of blood be cut off, for the currents of fluid, which during life permeated every part of bone cease, and the entire mass which has been affected by these changes must die."

We acknowledge that it is remarkably easy to conceive *all of that*, and to conceive more than that; we conceive what Dr. Beale does not state, perhaps not conceiving it himself, that *death* of the *germinal matter* in those *lacunae* might take place while the *full amount of blood* still *circulated around them*. After the death of the germinal matter however, we apprehend that the circulation would cease, even though all the vessels were open.

But more of this presently. Notice here, that caries and necrosis according to Dr. Beale, proceed from diametrically opposite conditions. Caries proceeds from too much pabulum; necrosis proceeds from want of pabulum. Again.

"Immediately around this (necrosed bone) the nutrient matter would flow more freely, but of course less regularly. In consequence the germinal matter of the neighboring lacunæ would grow much faster, and thus a number of granular cells result. These would even *eat away* as it were, but of course very slowly the dead bone, which soon becomes surrounded with such cells."—Idem p. 146.

So *necrosis produces caries* you perceive. Notice here also that the increase in germinal matter—cells, &c., around the necrosed bone, is not because the dead bone acts as an irritant or excitant but because of the increased amount of fluid that now passes *around* but once passed *through* the dead bone. The increase in quantity of nutrient fluid, necessarily determines the increase in amount of germinal matter, according to Dr. Beale.

Now we believe this theory of caries, and the explanation of the phenomena attending necrosis, to be absolutely fallacious in every particular. To be one series of unfounded assertions.

1. It is not philosophical; for philosophy investigates causes and effects and in assigning to any given effect its appropriate cause, it first proves its invariable antecedent relationship;

2. That *it is* appropriate. 3. That it is adequate to the production of this effect.

Now Dr. Beale has assigned too much nutriment as the *cause* of caries, and yet he has failed to establish its invariable antecedence, has nowhere shown that pabulum *is* really in *excess;* or *why* it should be in excess; or if really in excess, *why* it should produce this effect.

He assigns a cause and does not even attempt to show that it is always present; or that it is appropriate; that it is adequate. He does not even prove by the analogy of the operation of like causes in other tissues, that this effect should ensue from such a cause.

He proves nothing—he assumes everything—and then asks us to believe it. Which we emphatically decline to do, as the assumption of premises and deduction of conclusions therefrom, is not our idea of scientific reasoning.

It is in its very nature improbable.

The essence of this theory involves two propositions, neither of which we admit.

1. Proposition.—That germinal matter although too freely supplied with pabulum, yet makes a parasitical attack upon tissues already formed and literally eats them up.

If *too* freely supplied with pabulum why does it attack the formed tissue? Why does it with a surplus of nutrient material, yet wage a war of extermination against the very tissue it ought to produce and has been producing? Why with an abundance of food, does it devour its own offspring?

Is this cannibalism a characteristic of the germinal matter of bone only,—or is it true of germinal matter wherever found?

1.—Has germinal matter the power of decomposing formed tissue into its chemical elements, and recomposing these into pabulum. 2.—Or has it the power of transforming formed tissue into pabulum direct. 3.—Or has it the power of transforming formed tissue back into germinal matter direct?

If it *feeds* upon formed tissue it must do one of the three—which is it?

It may have such a power—but Dr. Beale has nowhere else assigned such to it; and we deny that he has the right to claim for germinal matter here, a power, which he has never granted it before.

We all know that tissues die, decay, moult incessantly, and are destroyed in exhibition of the different vital phenomena, and that they return to their ultimate elements, through chemical compounds formed in obedience to their chemical affinities; that these results of waste and decay are rapidly conveyed away by blood-vessels and eliminated by the proper organ; but that these tissues should be attached and devoured by the very *material* which *nature intended* for *their production*, is to our mind in the highest degree improbable.

Germinal matter therefore in the role of the parasite we cannot endorse.

2. Proposition—That germinal matter has the *power of transforming* but not the power of *refusing* to transform pabulum.

Now we assert that germinal matter has the power of refusing to transform pabulum, and this depends upon the "Law of *Limited* Vital Force."

If comparative anatomy teaches any one truth throughout its entire range, it is the truth of the limitation of vital force. Examine the whole animal creation, and you will find that, for classes—orders—tribes—families—genera—species—varieties and even for individuals, the amount of vital force is definite—determinate—circumscribed. Form—size—strength—intelligence, &c., are accurately and invariably apportioned to each, and by these are they characterized, distinguished and classified.

No superabundance of food or pabulum can change, or vary them except to an extent limited indeed.

This same law of limitaliou holds equally true in regard to the anatomical structure of all animals ; for the organs are limited in size—the tissues are limited in size, even down to their ultimate anatomical forms, such as the fibrillæ of muscle and ultimate nerve tubules, &c. They are all limited, all determinate in size and even the germinal matter itself never assumes a size larger than 1.1000 of an inch in diameter, no matter how great the amount of pabulum it receives.

Dr Beale grants it the limited size, but refuses it the power of limiting quantity.

The same power that limits the size of animals, size of organs, size of tissues, size of germinal matter, also limits the quantity of germinal matter produced, though there be excess of pabulum. Dr. Beale's theory that pabulum in excess, *necessarily* determines the rapid production of germinal matter, instead of *allowing* the *formation* of proper tissue, is not sustained by the history of the animal race.

The definite size of animals, of tissues, of even the minutest microscopical anatomical elements, give us the irresistible conclusion, that as the formed tissues are definite in size and quantity ; as germinal matter itself is definite in size, and as the tissues are formed from germinal matter, *then* must the quantity of germinal matter be definite also, and the amount of pabulum consumed definite, regardless of the amount present.

Then germinal matter *has* the *power* to *refuse* to transform pabulum.

Germinal matter *must* certainly have the power of refusing to transform pabulum when in excess, otherwise wherever over the whole system pabulum may be in excess, we must of course have all of the phenomena of caries, i. e. germinal matter living too fast and preying upon the surrounding tissues.

For Dr. Beale says germinal matter is the same everywhere, and of course the same effects must ensue where the same cause operates. But this theory is not sustained by the phenomena seen in the animal economy.

If germinal matter when living too fast, preys upon or consumes the formed tissue of bone, it certainly does not serve all tissues in the same way.

You remember in the lectures upon mucous and pus (Chamber's Renewal of Life,) we cited Dr. Sanderson of St. Mary's Hospital, London; Dr. Forster of Wurzburg; Buhl of Munich as authorities to prove that germinal matter could pass through the epithelial cells without injuring them; in fact, that purulent catarrh, diphtheritic membrane, &c., might exist, yet not the slightest change be found in the epithelial coat. The germinal matter that formed the mucus, pus, membrane, &c., must have come through the epithelial coat; and certainly did increase rapidly in quantity; and yet it *did not feed* upon the epithelial or formed tissue.

Why should it do so in bone?

Again—Buhl found that in some instances the epithelial coat was disorganized, broken up, eroded, &c., but he could not assert that the *germinal matter* had caused this, for in the previous cases it had left that coat intact, and to say that now it did destroy the coat would be a mere assertion. He could not prove that it fed upon the coat. Now in many of these cases the formed tissue, i. e. epithelium was perfect, yet the cases surely present the very symptoms characteristic of caries; that is, there was a rapid increase of germinal matter, and the formed tissue *should* by analogy have been preyed upon or eaten up, but it *was not.*

So germinal matter may live too fast;—increase too rapidly in quantity; pass *through* formed tissue, draw its nutrient fluid through formed tissue and yet, leave the *formed tissue perfect.*

Now why should it *consume* the formed tissue in bone? The opposite action in the two cases would seem to require explanation, but we defy you to find anything in Dr. Beale's work, which will harmonize the two. As one is the result of direct observation of several, and the other the mere theoretical assumption of *one man,* we do not hesitate which to reject.

(To be Continued.)

---

